# Treatment of malignant airway obstruction with Y-shape sigma stent loaded with I^125^ seeds installed via rigid bronchoscopy

**DOI:** 10.1186/s12890-024-03012-x

**Published:** 2024-04-24

**Authors:** Chunlong Lin, Hesong Huang, Lixia Song, Xixi Zhao, Jialing Zeng, Lun Li, Qilong Ge, Rui Li, Zhiyuan Wu

**Affiliations:** Department of Respiratory, Yueyang Municipal Hospital of Hunan Normal University, 263 Baling East Road, 414000 Yueyang, Hunan China

**Keywords:** Malignant airway obstruction, Y-shape sigma, Rigid bronchoscopy, Radionuclide, Safety and efficacy

## Abstract

**Purpose:**

To summarize and analyze the safety and efficacy of a Y-shape Sigma stent loaded with I^125^ in patients with inoperable malignant main airway obstruction.

**Methods:**

This study was approved by the Institutional Ethics Committee, and a written informed consent was obtained from each participant. A Y-shape Sigma stent loaded with I^125^ was placed under vision from rigid bronchoscopy. The primary endpoint was alleviation of symptoms and improvement of Karnofsky Performance Status (KPS) score, and the secondary endpoint was complications and technical success.

**Results:**

From November 2018 through June 2023, total 33 patients with malignant airway obstruction were palliatively treated by installing Y-shape Sigma stents loaded with I^125^. The airway lumen was immediately restored and the average airway opening significantly increased to 70 ± 9.4% after the procedure from baseline 30.2 ± 10.5% (*p* < 0.05). Average KPS score was improved from baseline 30.0 ± 10.0 to 70.0 ± 10.0 (*p* < 0.05) as well as PaO_2_ from baseline 50.1 ± 15.4 mmHg to 89.3 ± 8.6 mmHg (*p* < 0.05). The technical success rate of placing the stent in this study was 73%, and adverse events or complications including bleeding, I^125^ loss, and airway infection occurred during or after the procedure.

**Conclusion:**

Placement of Y-shape Sigma stents under vision from rigid bronchoscopy in the patients with malignant airway obstruction is feasible and it immediately alleviates dyspnea and significantly improves quality of life.

## Introduction

Main airway obstruction or stenosis occurs in patients with central lung cancer or the patients with pulmonary metastases from other malignances including esophageal carcinoma and malignant lymphoma, which leads to endotracheal or endobronchial obstruction and/or extrinsic compression [1, 2]. While the ablative surgical procedure is often used to destroy tumor tissues of endobronchial obstruction, endotracheal or endobronchial stent placement has been used as an alternative intervention for the patients with malignant main airway obstruction [3–6]. However, stent restenosis occurs in some of the patients because of malignant infiltration through the meshes into the lumen or out-growth at the ends of the stent [7, 8]. To overcome this complication, airway stents loaded or coated with radioactive isotopes have been developed for preventing neoplastic re-growth-associated restenosis, which was similar to the application of radioactive stents for the treatment of patients with malignant esophageal or biliary obstruction [9, 10].

The iodine-125 (I^125^) is a commonly used radionuclide with a half-life of 60 days. I^125^ seeds provide continuous gamma rays in a low dose (a maximum energy of 0.035 Million Electron Volts), which could kill tumor cells by synchronizing the cancer cells to radiosensitive G_2_-M phase [11], but allow normal tissues to repair the sublethal tissue damage [12]. Thus, I^125^ seeds are appropriate to be used in brachytherapy for variety types of malignances. In the current study, a Y-shape Sigma stent loaded with I^125^ seeds was applied to the patients with unresectable central lung cancer complicated with main airway obstruction, and here, we present the therapeutic outcome and procedure-associated complications.

## Materials and methods

### Patient enrollment

This was a single center study. Patients who visited our hospital from November 2018 through June 2023, and met the following criteria were enrolled into this study. 1). Patients with pathological diagnosis of lung cancer. 2). The score of Karnofsky performance status (KPS) was < 60, and survival prognosis was > 3 months. 3). Patients had malignant tumor-associated tracheal or main bronchial obstruction or stenosis. 4). Patients had progressive or remained no change in lung cancer following chemotherapy or radiotherapy. 5). Patients had recurrent lung cancer after surgical resection. 6). Surgical resection of the lung cancer was not an option for the patients due to high age or abnormal heart and lung functions. 7). Patients who rejected to have surgical resection of the lung cancer. 8). Platelet count was > 50 × 10^9^/L. 9). Patients or legal guidance signed an informed consent form, and willing to participate the study.

### Endpoints and definitions

The primary endpoint was to assess the grade of restoring airway patency and airway lumen inner diameter after the procedure in comparison with the baseline, alleviation of symptoms, and improvement of PaO_2_ and lung function. In addition, complications during and after the procedure were also analyzed as secondary endpoint.

### Materials and procedure

Before the procedure, routine blood test panel, blood biochemistry, coagulation function, ECG, Karnofsky performance status, and chest CT examination were performed in all patients. The iodine 125 (I^125^) seeds were obtained from a commercial source (Junan Co., Niingbo city, Zhejiang Province, China). Dose of I^125^ seeds for each patient was determined by evaluating the tumor size, number and location of the tumor(s), and distance of the tumor from vessels by regular and 3D enhanced chest CT scan. Based on the evaluation, chest CT angiogram followed by pulmonary artery embolism were performed in some of the patients to prevent bleeding during and post-procedure. Stent type and size were determined prior to the procedure based on the examination of airway 3D CT imaging as well as bronchoscopy. Y-shape stents (recyclable Sigma CZTS type) were provided by Huaian Sigma Medical Industry Co, Ltd. (Huaian city, Jiangsu Province, China). The tracheal stent was 16 mm in diameter and 50–70 mm in length, the left main bronchial limb of the stent was 12–13 mm in diameter and 30–45 mm in length, and the right main bronchial limb was 13–15 mm in diameter and 15 mm in length.

The procedure was performed by experienced physicians who were required to wear lead bib, lead gloves, lead glasses, and lead aprons. After successful general anesthesia and mechanical ventilation, a guidewire was placed through the stricture of lesion under rigid bronchoscope followed by delivering the stent sheath. The stent, which had been pre-loaded with radioactive I^125^ seeds, was then delivered through the sheath. Once the stent was expanded at the target location under the guidance of the sheath, the sheath was immediately withdrawn.

### Statistical analysis

Continuous variables were expressed as mean ± standard deviation (SD). Categorical variables were described as frequencies and percentages. SPSS26.9 software was used to perform statistical analysis. Student’s *t* test was used to compare the mean ± SD of therapeutic outcomes. *P* < 0.05 was considered as significant.

## Results

### Patients and demographic information

From November 2018 through June 2023, total 33 central lung cancer patients with varying grade of airway obstruction were treated with Y-shape Sigma stents loaded with radioactive I^125^ seeds. Of them, 19 were male and 14 were female, and majority of them were over 50 years old. Nearly half of the tumors were found in trachea (36.4%) or carina of trachea (12.1%), and the other half of the tumors were in the main bronchi with dominantly on the right bronchus (39.4%). Pathological examination indicated that 63.6% were squamous cell lung cancer, and the rest 36.4% were adenocarcinoma. All patients were at stage II or later stages, and 31 of them (94%) had grade IV or V airway obstruction with severe difficulty in breathing (Table 1).

**Table 1:** Demographic features of the patients (*N* = 33)

	Number	Percentage (%)
**Gender**		
Male	19	57.6
Female	14	42.4
**Age**		
≤ 50	3	9.1
51–60	9	27.3
61–70	12	36.4
> 70	9	27.2
**Smoking**		
Yes	18	66.2
No	15	33.8
**Comorbidity lung diseases**		
COPD	20	60.6
Bronchiectasis	5	15.2
Silicosis	2	6.1
Tuberculosis	6	18.1
**Location of lung cancer**		
Trachea	12	36.4
Carina of trachea	4	12.1
Left bronchi	4	12.1
bronchi	13	39.4
**Cell type of the tumors**		
Squamous cancer	21	63.6
Adenocarcinoma	12	36.4
**Combined with chemotherapy**		
Yes	16	48.5
No	17	51.5
Grade of airway obstruction *		
III	2	6.1
IV-V	31	93.9
**TNM stages**		
Stage II	6	18.2
Stage III	7	21.2
Stage IV	20	60.6
**KPS score prior to the treatment**		
50–59	9	27.3
40–49	14	42.4
30–39	10	30.3

*Severity of the tumor-associated airway obstruction was determined as previously described [Wang, 2021]. Grade I: ≤ 25% airway obstruction with mild coughing; Grade II: 26–50% airway obstruction with mild coughing and short of breath; Grade III: 51–75% airway obstruction with moderate coughing and short of breath; Grade IV: 76–90% airway obstruction with severe chest tightness, short of breath, and difficulty in breathing; Grade V: 91–100% airway obstruction with very severe chest tightness, short of breath, and difficulty in breathing

### Primary and secondary endpoints

**Fig. 1:**
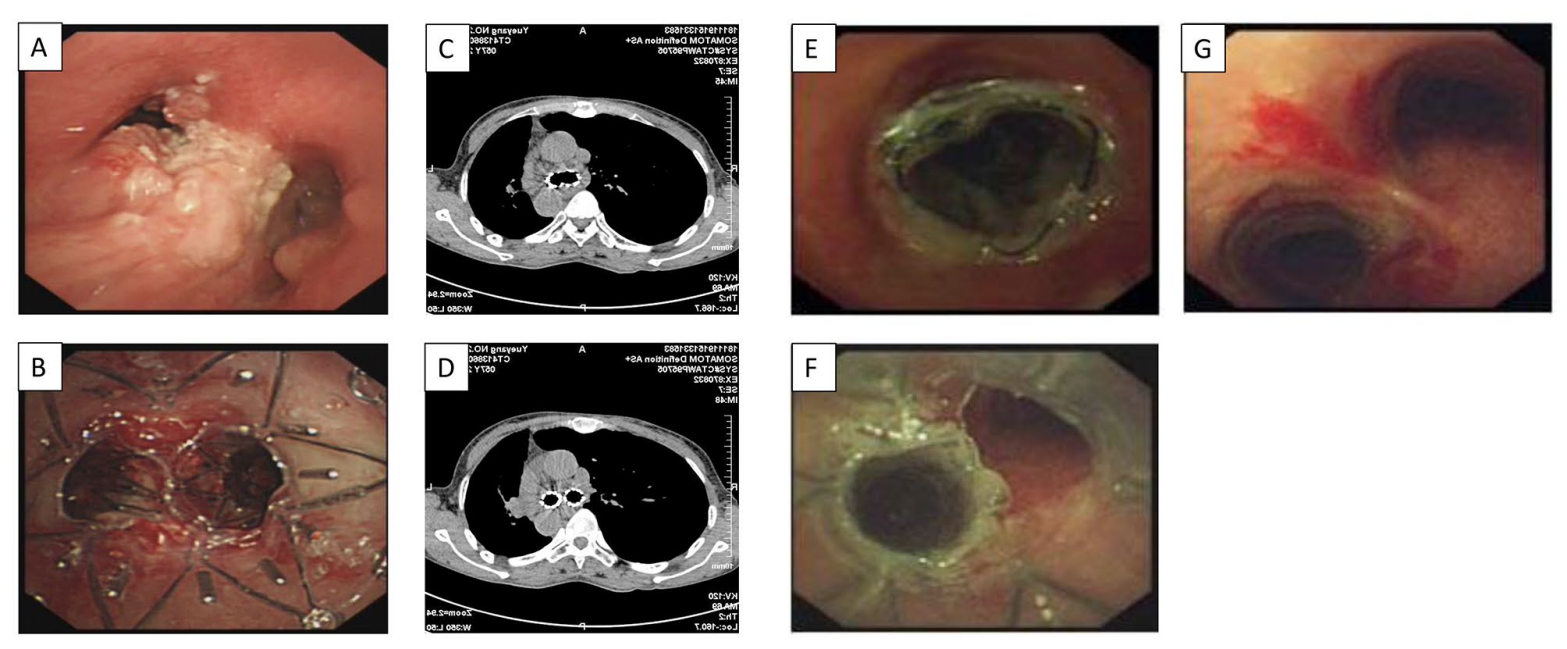
Images of central lung cancer-associated airway obstruction and its treatment with a Y-shape Sigma stent plus I^125^brachytherapy. **Panel A**: Image of airway obstruction by a malignant tumor located at carina of trachea. **Panel B**: Image of Y-shape Sigma stent, which was installed at trachea as well as left and right main bronchi following debulking resection of the lung cancer at carina of trachea. **Panel C** and **D**: Follow-up images of chest CT after one week of the stent installation. **Panel E** and **F**: Images under rigid bronchoscopy after one month of installment of the stent loaded with I^125^. **Panel G**: Image of carina as well as left and right main bronchi under rigid bronchoscopy after removal of stent at 6 months follow-up

As shown in Table 2, tracheal lumen opening increased from baseline of 30.2 ± 10.5% to 70 ± 9.4% right after the procedure (*p* < 0.05), and continuously increased at 1, 3, and 6 months after the procedure (79.2 ± 9.4%, 80.4 ± 3.9%, 81.4 ± 6.6%, respectively, *p* < 0.05 compared to 70 ± 9.4% at 0 month). Representative images of one representative case were shown in Fig. 1. Before the procedure, the airway was almost completely obstructed by the tumor at the carina of trachea (Fig. 1A). After debulking ablative resection of the tumor under rigid bronchoscopy, a Y-shape Sigma stent, which was pre-loaded with I^125^ seeds, was placed in the location of tumor growth (Fig. 1B). After one week of stent installation, images of chest CT (Fig. 1C and D) indicated the airway remained open by the stent. Moreover, at one-month follow-up, bronchoscopy examination (Fig. 1E and F) indicated that the tumor was significantly reduced in size with nearly 100% opening of the airway. After 6 months of the procedure, significant remission of the tumor was observed, and the airway remained completely opening even if the stent was removed (Fig. 1G). Consistent with the outcomes of airway opening, average KPS score was improved from baseline of 30.0 ± 10.0 to 70.0 ± 10.0 (*p* < 0.05), PaO_2_ from 50.1 ± 15.4 mmHg to 89.3 ± 8.6 mmHg after the procedure (*p* < 0.05, Table 2). Furthermore, both KPS score and PaO_2_ were also continuously improved during the 6 months follow-up evaluation (*p* < 0.05, Table 2).

**Table 2:** Therapeutic outcomes of Y-shape Sigma stent pre-loaded with ^125^I seeds

	Airway opening (%)	KPS	PaO_2_ (mmHg)
Baseline	30.2 ± 10.5	30.0 ± 10.0	50.1 ± 15.4
After the procedure 0 month 1 month 3 months 6 months	70.3 ± 9.4* 79.2 ± 9.4*^#^ 80.4 ± 3.9*^#^ 81.4 ± 6.6*^#^	70.0 ± 10.0* 72.5 ± 8.7* 73.6 ± 7.7* 72.6 ± 8.4*	89.3 ± 8.6* 90.2 ± 7.8* 91.4 ± 6.5* 89.9 ± 7.2*

After 1 to 6 months of the procedure, patients had significant improvement in airway opening, respiratory functions, and partial pressure of oxygen in arterial blood. *p < 0.05 compared to those of baseline, # *p* < 0.05 compared to 0 month

The procedure of Y-shape Sigma stent placement was performed by experienced Pulmonologists with some adverse events encountered during the procedure. Moreover, incidence of various complications after the procedure was also observed in this study. As shown in Table 3, all 33 patients had varying grades of cough and/or wheezing, which was mainly due to airway edema or hypersensitive reaction to metallic stent; 31 patients had difficulty in coughing phlegm up; 19 patients had ulcer of the trachea mucosa due to the tissue damage by stent or radiation; 14 patients had mild to moderate chest pain; 6 patients had fever and hemoptysis; 5 patients had airway infection; 5 patients had secondary airway stenosis due to hyperplasia of granulation tissue along the stent; 4 patients had temporal dysphonia or pharyngitis; 3 patients had tracheal adventitia rupture and out-growth of the tumor towards mediastinal space; and 2 patients had re-stenosis of the stent due to infiltration of the tumors into stent mesh or out-growth at the ends of stent.

**Table 3:** Post-procedure complications and their potential causes

Complication	# of cases	Potential cause(s)
Cough, wheezing	33	Metal hypersensitivity reaction, Reinke’s edema; excessive mucus or phlegm, infection
Difficulty in phlegm excretion	31	Stent-associated airway tissue damage, lumen expansion, or airway mucous membrane irritation
Ulcer of the tracheal mucosa	19	Tissue damage by radiation or stent
Chest pain	14	Airway inflammation, endothelial cell injury and ischemia following the procedure; over-size stent resulted in over expansion of airway lumen
Fever and hemoptysis	6	Tissue injury by the ends of stent, or compression of the peripheral blood vessels by stent that could result in vessel wall necrosis and rupture
Airway infection	5	Stent restenosis caused airway obstruction, difficulty in phlegm excretion
Secondary airway stenosis	5	Granulation tissue hyperplasia after the stent implantation
Temporal dysphonia or pharyngitis	4	Mucosal edema
Tracheal adventitia rupture followed by tumor infiltration into the mediastinal space	3	Maloperation of stent placement procedure
Stent restenosis	2	Tumor re-growth due to insufficient energy of ^125^I or hyperplasia of granulation tissue at the ends of the stent or infiltration through stent mesh

## Discussion

Late-stage central lung cancer and enlarged malignant lymph nodules often result in main airway obstruction or stenosis. While systemic chemotherapy is a standard treatment for late-stage malignancies, chemotherapy does not immediately restore airway patency for the patients with main airway obstruction. In contrast, recanalization of the airway by placing a stent in the main airways could result in immediate relive of the airway obstruction and restore the airway patency [13]. However, due to continuous growth and infiltration of the malignancies into airway lumen, airway restenosis could occur in up to 45% of the patients who received airway stent placement [7]. Thus, stent loaded with radioactive isotopes has been developed to overcome this complication. In the current study, total 33 patients with main airway obstruction due to the neoplastic of squamous lung cancer or adenocarcinoma were successfully treated with Y-shape Sigma stent, which was pre-loaded with I^125^ and installed under the vision from rigid endoscope. Consistent with the previous reports [3, 4, 13], installation of Y-shape Sigma stents benefited the patients by significant improvement of the quality of life through immediate re-opening the main airways although the procedure was a palliative treatment for the late-stage cancer patients.

Airway stent placement has been used as a palliative therapy with the advantage of rapid restoring patency for patients with major airway obstruction [1, 8]. Unfortunately, however, it has been reported that stent restenosis occurred within 3 months due to the tumor in-growth in 24% or over-growth in 21% of the patients [7]. Therefore, combination of stent and brachytherapy with radioisotopes has been developed in the treatment of various malignant lumen obstructions including lung cancer, esophageal cancer, or malignant biliary tumor [3, 9, 10, 14]. Application of the stent loaded with radioactive seeds could result in not only rapid achievement of lumen reopening, but also long-term control of the tumors by continuous brachytherapy. Iodine-125 (I^125^) is the most commonly used radioactive isotope for brachytherapy in that I^125^ is a radionuclide with a half-life of 60 days, and it emits low-dose gamma rays with a maximum energy of 0.035 Million Electron Volts (MeV). Therefore, in the current study, I^125^ was pre-loaded on the surface of Y-shape Sigma stent and installed in the location of stenotic upper airways caused by central lung cancer. While the overall survival was not observed and analyzed in our study, quality of life in all 33 patients was improved significantly, and some of them had good tumor control and symptom alleviation even after stent removal. These preferable outcomes may be attributed to the continuous inhibition of tumor growth by the accumulated low dose I^125^ and continuous emission of gamma rays at the location of malignancies. In addition, the efficacy of stent plus I^125^ seeds treatment in our study suggested that both squamous cell lung cancer and adenocarcinoma are radiosensitive to the low-dose-rate I^125^ brachytherapy.

CT-guidance has been used in the placement of stent with or without radioactive seeds for the treatment of malignant airway obstruction caused by lung cancer or esophageal cancer [3, 15]. In the current study, however, rigid bronchoscope was used to place Y-shape Sigma stent coated with I^125^. Compared to the CT-guided placement of stent and radioactive seeds, the procedure under vision from the rigid or flexible bronchoscope has the following advantages. First, the stent and radioactive seeds were accurately placed at the stenotic segment of airway under the vision of endoscope. Second, both patient and doctors exposed less radiation during the procedure. Third, precise dose of seeds and accurate location of stent placement could lead to better therapeutic outcome than traditional brachytherapy. Fourth, the radioactive stent could be taken out under the bronchoscope at the end of brachytherapy or anytime if it needs to be removed.

Stent placement and radiation therapy could have various adverse effects such as bleeding and coughing. In the current study, majority of the patients had experienced cough, wheezing, and difficulty in phlegm excretion after the procedure of stent placement, which was mainly due to hypersensitivity of airway mucosa to the metallic stent, Reinke’s edema, or airway infection. Radiation or metallic stent also led to ulcer of the tracheal mucosa by direct damage of the tissues. Although airway infection could exist before the stent placement procedure in some of the patients with upper airway obstruction, airway infection rate after the procedure was 15% (5/33) in our study, which was slightly higher than the 5–10% in the previous reports [4, 6, 16], suggesting that the risk of airway infection following the procedure of stent placement loaded with radioactive isotope be emphasized.

In the current study, although the technical success rate of the stent placement through the rigid bronchoscopy was high, there were some problems or unexpected events occurred during the procedure. As shown in the Table 4, bleeding was one of the most encountered events (36%, 12/33) in this study followed by un-satisfaction rate of the stent was placed (4/33) or incomplete expansion of the stent (3/33). We believe the main cause of bleeding was over-vascularized tumor blood vessels in the central type lung cancer, mechanic tissue damage by the stent metal, and necrosis of the tissue in response to the radioactive I^125^ seeds. In addition, stent migration or displacement occurred in 2 cases, loss of the coated I^125^ seeds occurred in 2 cases due to repeated expanding and collapsing the stent, and bronchial rupture occurred in 2 cases because of placing the whole Y-shape stent into one side of main bronchus by mistake. Nevertheless, all 33 patients had immediate relief of dyspnea following the procedure, which attributed to the Y-shape Sigma stent that reopened obstructive trachea and main bronchi.

**Table 4:** Adverse events encountered during the procedure

Adverse events	# of case	Potential cause(s)
Incomplete expansion of the stent	3	Stent was stiff, tumor suppression
Unsatisfied location of the stent	4	The two limbs of Y-shape stent were too short or in same length, difficult to insert or pass through the stenotic location
Stent migration	2	Enlarged airway lumen after ablative surgery, size of the stent was small
^125^I seeds loss	2	The coating was hard and brittle, and torn out due to repeated expanding and collapsing
Guide wire broken	1	Incorrect operation
Rupture of coating	3	Incorrect operation
Rupture of bronchi	2	Whole stent was placed into only one side of the main bronchus
Bleeding	12	Tumor angiogenesis and its blood supply vessels were not blocked by embolizing pulmonary artery before the procedure
Pneumothorax	1	Rupture of a bronchus lead to mediastinal pneumothorax

There were limitations in this study. First, only the patients treated with stent and brachytherapy was analyzed in this study, which was lack of comparison with those who were treated by standard chemotherapy or radiotherapy. Second, long-term follow-up was not conducted, and overall survival rate was not analyzed due to the limited sample size. Third, findings of the current study remain to be further evaluated in a randomized prospective and multicenter cohort study. Nevertheless, a personalized protocol on radiation dose of I^125^ and individualized stent selection by the grade of airway obstruction and location of the malignancies in this study seemed to be effective with preferable outcomes in most of the participants.

Taken together, our study demonstrated the successful application of Y-shape Sigma stent loaded with radioactive I^125^ seeds for palliative treatment of the main airway obstruction by unresectable central lung cancer. The procedure of airway stent installment could be performed under the vision from rigid bronchoscope with high rate of technical success rate. Restore of airway patency by the Y-shape Sigma stent and the low-dose-rate brachytherapy with radioactive I^125^ seeds could benefit the patients through instant alleviation of dyspnea and significant improvement in the quality of life. Clinical trials could be taken in the future to validate its efficacy and tolerability in patients with main airway obstruction caused by central lung cancer.

## Data Availability

No datasets were generated or analysed during the current study.
